# Safety and efficacy of early atropine injection for dobutamine stress cardiac magnetic resonance: a single center experience

**DOI:** 10.1186/1532-429X-13-S1-P127

**Published:** 2011-02-02

**Authors:** Vincent Woo, Stanley Lau, Gerald M Pohost

**Affiliations:** 1 Harbor-UCLA Medical Center, Torrance, CA, USA; 2 University of Southern California, Los Angeles, CA, USA; 3 Keck School of Medicine, University of Southern California, Los Angeles, CA, USA

## Introduction

Dobutamine is an effective pharmacologic stress agent commonly used in cardiac stress testing, but has several well documented adverse effects including serious arrhythmias and myocardial infarction. The most common dobutamine protocol is to infuse an increasing dose of 10 µg/kg followed by 20, 30, then 40 µg/kg until target HR is achieved. Atropine may be added after the 40 µg/kg infusion if target heart rate is not achieved. An alternative strategy for dobutamine infusion involves earlier atropine administration, i.e. infusion of atropine after the 10, 20, or 30 µg/kg doses of dobutamine. In the echocardiography literature this strategy has been shown to have a better safety profile while maintaining the ability to achieve target heart rate. Dobutamine stress CMR may benefit from adopting a similar protocol.

## Purpose

The objective of this study is to assess the safety and efficacy of the addition of early atropine injection to dobutamine for stress CMR.

## Methods

This is a single center, retrospective study involving 168 patients undergoing early-atropine dobutamine stress CMR for the evaluation of chest pain. All patients underwent a hybrid stress CMR protocol with evaluation of wall motion, perfusion, and late gadolinium enhancement. Demographic and stress CMR data are gathered from an electronic medical records system. The stress CMR data that were collected included the doses of dobutamine and atropine administered, the time of atropine infusion, the heart rate and blood pressure responses, and the incidence of adverse effects. All data are entered into a Microsoft Excel spreadsheet, where statistical analyses were performed.

## Results

**Figure 1:**
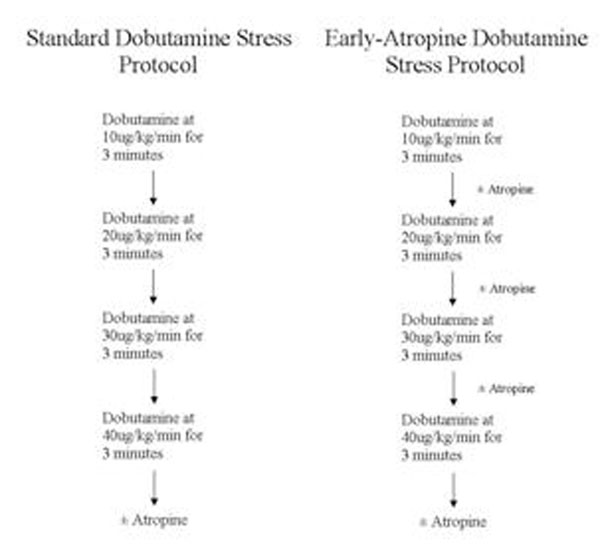


The mean age of the patients undergoing CMR was 70.8 ± 7.6 years, with 91 females and 77 males. 156 patients were Asian, 8 were Caucasian, and 3 were Hispanic. 128 patients had hypertension, 96 had hyperlipidemia, and 46 had diabetes. 53 patients had documented coronary artery disease. Patients received an average of 8.5 ± 4.8 mg of dobutamine and 0.26 ± 0.19mg of atropine during the examination. Of 168 patients, 158(94%) achieved target heart rate or85% of maximum predicted. In our population, 14 patients were positive for ischemia. Of 168 examinations, 167 were considered of diagnostic quality. Three patients were hypotensive during the examination, while 1 was hypertensive 3 reported chest pain, and 1 reported dizziness. Tables 1-3, figure 1.

**Table 1:** Patient demographics

Age	70.8 ± 7.6 years
Gender	45.8% males
Hypertension	76.2%
Hypercholesterolemia	57.1%
Diabetes	26.2%
Coronary Artery disease	31.5%
Past Myocardial Infarction	9.5%
Past Percutaenous Coronary Intervention	10.7%
Past Coronary Artery Bypass Graft	11.9%
Congestive Heart Failure	4.2%

**Table 2:** Hemodynamic response

	Rest	Stress
Heart Rate (BPM)	71.2 ± 12.1	130.1 ± 12.6
Systolic Blood Pressure (mmHg)	142.8 ± 19.3	154.8 ± 85.3
Diastolic Blood Pressure (mmHg)	80.6 ± 12.3	85.3 ± 15.1

**Table 3:** CMR results

CMR Results	
Positive Stress CMR	

85% maximal HR reached	10
85% maximal HR not reached	4

Negative Stress CMR	

85% maximal HR reached	153
85% maximal HR not reached	1

## Conclusions

The early-atropine, dobutamine stress CMR test can be performed with an excellent safety profile and a lower dose of stress agent compared with the standard late-atropine strategy.

